# Electrochemical Detecting Lung Cancer-Associated Antigen Based on Graphene-Gold Nanocomposite

**DOI:** 10.3390/molecules22030392

**Published:** 2017-03-02

**Authors:** Zheng Wei, Junping Zhang, Aihua Zhang, Yanchun Wang, Xiaoping Cai

**Affiliations:** 1 Department of Oncology, Henan Academy institute of Traditional Chinese Medicine, Zhengzhou 450004, Henan, China; questwz@163.com (Z.W.); zhangjunping888@163.com (J.Z.); zhangaihuatcm@163.com (A.Z.); 2 Department of Traditional Chinese Medicine, Henan Province People’s Hospital, Zhengzhou 450004, Henan, China; wang88982@126.com

**Keywords:** label-free immunosensor, neuron-specific enolase (NSE), signal-enhanced, electrochemical immunosensors, graphene

## Abstract

Using a Au nanoparticle/reduced graphene oxide composite (AuNP-RGO), a signal-enhanced electrochemical immunosensor without label was created to detect neuron-specific enolase (NSE). Furthermore, an environmentally-friendly method was developed to prepare AuNP-RGO by employing chitosan (CS), which served as reducing and stabilizing agent. We showed that the sensitivity of the immunosensor designed in this report was remarkably enhanced because of the numerous active sites in the sensor provided by the AuNP-RGO nanostructure. For the quantification of NSE, the immunosensor exhibited a positive linear relationship with the concentration in the range of 0.1 to 2000 ng/mL, where the limit of the detection was 0.05 ng/mL.

## 1. Introduction

Human lung cancer—consisting of the two main categories small cell lung carcinoma and non-small cell cancer—is the major factor of cancer death rate all over the world [1,2]. Around 15%–20% of the new cases of lung cancer are caused by small cell lung cancer, where most of these cases have been diagnosed with the serious illness. Neuron-specific enolase (NSE)—one type of neuroendocrine enzyme—can serve as the assumed serum marker of small cell cancer and exhibits a remarkably diagnostic specificity and sensitivity [3,4,5,6,7,8,9]. Recently, the probability of the associated NSE serving as a highly-expressed cancer marker of non-small cell lung carcinoma has been reported. In general, small cell lung cancer becomes suspicious when the level of total NSE in serum goes beyond 35 ng/mL. Moreover, it is demonstrated that the measurement of the serum concentrations of NSE might be beneficial to predict the response to therapy earlier so as to improve the individual control of the disease and the prognosis of small cell lung cancer.

Lung cancer has been considered as the uncontrolled increase of unusual cells in single or both lungs. Normal functionalities have been lost in these abnormal cells, which will stop developing and differentiating into healthy lung tissue. Then, these disordered cells will generate massive tissue considered as the tumour, which can hinder the transport of oxygen to the body though the blood circulation system from the lungs. Among all the carcinoma death of both females and males globally, lung cancer ranks the first. For instance, about 26% and 31% of cancer death in women and men was attributed to lung cancer in 2008. The risk of tumour cell metastasis and cancer recurrence remains, even under therapy. Non-small cell lung carcinoma is known as one of the two most usual kinds of lung cancer based on the American Cancer Society, which is responsible for around 80% of all new cases. Consequently, an intensive monitoring strategy for non-small cell lung carcinoma and the development of proper screening approaches such as NSE are urgently needed.

The function of the immunosensor is dependent on the antibodies, which can react with the corresponding antigens and the quantification is normally completed by measurements of the particular activity of the label, such as bioluminescence [10], fluorescence [11], enzyme or chemical amplification [10], radioactivity [12], electrochemical response [13,14], surface plasmon resonance [15], and the influence on the quartz crystal microbalance [16]. According to the immunobioluminescent tests [17,18], mass spectrometry [19], and two-dimensional (2D) differential in-gel electrophoresis [20], several approaches to the detection of NSE have been reported. However, complicated instrumentation and sophisticated and time-consuming pretreatment of sample are generally necessary in these methods. Consequently, electrochemical immunosensors have become a promising analytical method to detect antibody and antigen in the past decades, because of their high sensitivity, relatively low-cost, and quick analysis time [21,22,23]. Recently, nanoparticle-based electrochemical immunosensors have attracted intensive attention with the development of nanotechnology, due to their special electrical, optical, catalytic, and thermal properties [24,25,26].

Graphene, a 2D sp^2^ carbon networking material with a single-atom thickness, has attracted intensive interest because of its significant mechanical, electrical, and thermal properties. Thus, these distinct properties of graphene have been applied in the design of versatile devices including electrochemical resonators, ultrasensitive sensors, and field-effect transistors. It has been demonstrated that graphene oxide (GO) and reduced graphene oxide (RGO) are promising in the applications of cellular imaging, biosensor fabrication, and drug delivery because of their easy synthesis, high accessible surface area, good water dispersibility, and biocompatibility. Recently, it has been reported that electrodes modified with RGO in the presence of interfering agents exhibit a remarkable electrochemical biosensing capacity in detecting electroactive biomolecules compared to bare electrodes and carbon nanotube-based electrodes. In distinguishing mixed species, the remarkable behavior exhibited by surfaces modified with RGO was ascribed to the significant electrocatalytic activity, owing to the large surface area and high density of the edge-plane-like defects in RGO sheets, which induce the quick heterogeneous transfer. Moreover, it has been demonstrated that gold nanoparticles are the predominant alternative to develop biosensors because of their high surface/volume ratio and distinctive and specific mechanical and electrical properties [27,28,29].

Herein, we synthesized a nanostructured sensing film with Au nanoparticle/reduced graphene oxide (RGO-AuNPs) nanocomposite and created a signal-enhanced immunosensor for NSE without a label. Specially, a significant activity was observed in the RGO-AuNPs nanocomposite, which might be useful in catalyzing the reduction of H_2_O_2_. Consequently, it indicated that the immunosensor designed in this work exhibited a remarkable capacity and high sensitivity. Then, the immunosensor was employed in the determination of the concentration of NSE in serum samples, where the obtained results were in accordance with those observed in the conventional clinical process.

## 2. Results and Discussion

**Figure 1:**
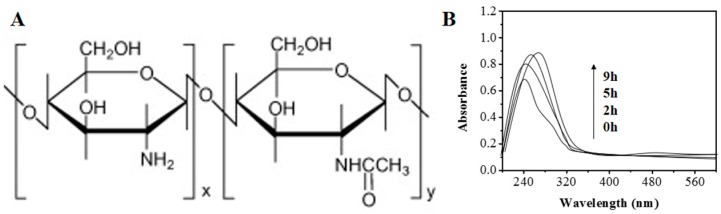
(**A**) Chemical structure of chitosan (CS); (**B**) UV-vis absorption spectra of the reduced graphene oxide (RGO) recorded at various times.

Chitosan (CS) is a natural biodegradable biomaterial that has been intensively employed as drug carrier, wound dressing, scaffold for tissue engineering, green packaging, and anti-adhesion materials. Moreover, it has also been used as a stabilizing agent for RGO suspensions due to its nontoxicity, hydrophilicity, and the covalent interactions with RGO [30]. As shown in Figure 1A, GO contains numerous carboxylic and hydroxyl groups, where CS contains plenty of amino groups in its macromolecular chains. In accordance with other reducing agents, including benzylamine [31], tea polyphenol [32], and hydrazine [33], the reduction of GO is assumed to be caused by the direct redox reaction between GO and the -NH_2_ groups in CS. Interestingly, the RGO dispersion is highly stable, which indicates that CS could adequately stabilize RGO. CS contains various functional groups including -OH and -NH_2_ or O=C-NH_2_ on its macromolecular chains, which can generate hydrogen bonds and electrostatically interact with the rest of the oxygen-based groups in RGO. Then, the CS macromolecules could be adsorbed onto the RGO nanoplates by these interactions. Thus, the RGO nanoplates could be stably dispersed in water.

Moreover, CS could also reduce GO under a relatively low temperature. UV-Vis absorption spectroscopy was employed to monitor the GO reduction process. In Figure 1B, it is obvious that the maximum absorption peak (λ*_max_*) of the GO suspension varied to 282.5 nm from 241.5 nm gradually, where the absorption intensity significantly raised. This indicated that the GO was reduced. Moreover, the electronic conjugations and the structure of GO was restored [34]. Fernandez-Merino et al. reported that the level of the reduction could be evaluated by using the red-shift of λ_max_ [35]. However, no changes in the absorption intensity and λ_max_ were observed when the reaction was performed for a longer time. Thus, the reaction time was set to be 9 h in the following experiments. In addition, CS could serve as the stabilizing agent as well as the reductant because the as-prepared RGO could be stably dispersed in water.

**Figure 2:**
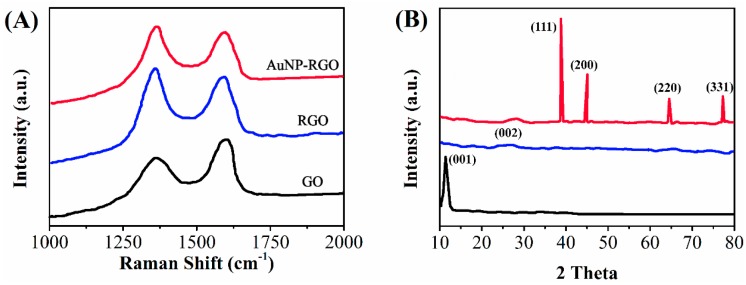
(**A**) Raman spectra and (**B**) XRD patterns of GO, RGO, and RGO/AuNP (Au nanoparticle/reduced graphene oxide) nanocomposite.

Raman scattering is an effective instrument with high sensitivity for the characterization of carbon-based materials. The Raman spectra of GO, RGO, and RGO/AuNP is shown in Figure 2A. For GO, two feature bands at 1308 cm^−1^ (D band) and 1601 cm^−1^ (G band) were observed in the spectrum of GO, which are ascribed to the sp^3^ and sp^2^ carbon hybridization, respectively [36]. Compared with GO, the I_D_/I_G_ ratio of RGO (1.34) is significantly higher after electrodeposition, which suggests that the mean size of the sp^2^ sites increases. According to the references, the reduction has been considered to take place [37,38]. Except for a small upshift in the G-band (from 1601 to 1604 cm^−1^), a similar scattering profile was observed in the spectrum of RGO/AuNP with RGO, which is caused by the doping of Au nanoparticles on the RGO sheets [39,40].

XRD is employed to analyze the crystalline structure and the interlayer changes of GO, RGO, and RGO/AuNP. The XRD data is illustrated in Figure 2B. A characteristic peak (001) at 11.0° is observed in the XRD pattern of GO as expected, where the value of *d*-spacing is 0.80 nm [41]. Nevertheless, for RGO, a nearly indistinct XRD pattern was observed, indicating that GO was reduced. A small peak located at 22.9° is caused by the stacked graphene layers of RGO [42]. For RGO/Au nanocomposite, the diffraction peaks at 39.4°, 46.1°, 67.5°, and 81.4° are ascribed to the (111), (200), (220), and (311) planes of the face-centered-cubic (fcc) crystallographic structure of Au (JCPDS 4-0783), respectively. This demonstrates that Au nanoparticles have been successfully electrodeposited.

**Scheme 1:**
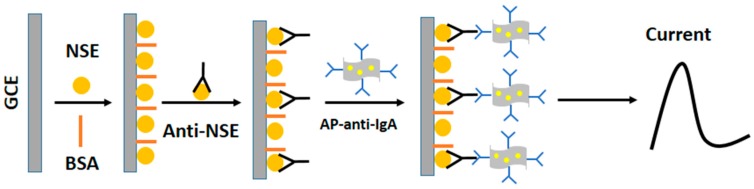
Schematic representation of the designed electrochemical immunosensor for neuron-specific enolase (NSE) detection. BSA: bovine serum albumin.

Using alkaline phosphatase (AP)-conjugated secondary antibody to label RGO/AuNP, AP-anti-IgG/RGO/AuNP was designed for immunoassay. The AP-anti-IgG/RGO/AuNPs exhibited highly catalytic activity toward the hydrolysis of α-naphthyl phosphate (α-NP), leading to a dual signal amplification of nanoprobe for the detection of low-concentration target. The scheme of the immunosensor is described in Scheme 1.

**Figure 3:**
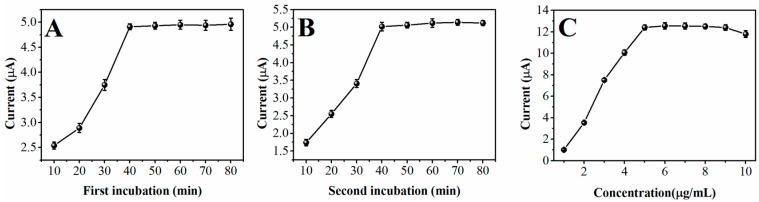
Effect of peak current on the incubation time of (**A**) the first incubation step and (**B**) the second incubation step with 100 ng/mL NSE; (**C**) incubation concentration of anti-NSE, when changing one parameter and keeping the others at the best conditions. The standard deviations—calculated from three different tests—are described by the error bars.

Moreover, the incubation time of the competitive immunoreaction as well as binding the AP-anti-IgG/AuNP-RGO to the anti-NSE antibody was optimized. In Figure 3A,B, it is obvious that the differential pulse voltammetric (DPV) peak current increased significantly when incubation time increased. However, the current tended to be steady after an even longer incubation time was performed. Hence, 40 min was considered as the optimal incubation time in the subsequent incubation steps. Moreover, a competition between the target NSE and the NSE domains on the surface of the electrode to bind the anti-NSE antibody in the incubation solution took place in the competitive immunoreaction. Hence, the content of anti-NSE antibody in the incubation solution was crucial for the competitive immunoassay. NSE/glassy carbon electrode (GCE) were incubated in the anti-NSE antibody solutions in various concentrations to optimize the concentration of the anti-NSE antibody. In Figure 3C, an increase in the peak current was observed when the concentration of anti-NSE increased, where the peak current tended to be plateau at 5 μg/mL. This indicated that all the sites available for the recognition of the anchored NSE matched with the anti-NSE antibody. Consequently, the anti-NSE antibody with a concentration was utilized in the first incubation step.

**Figure 4:**
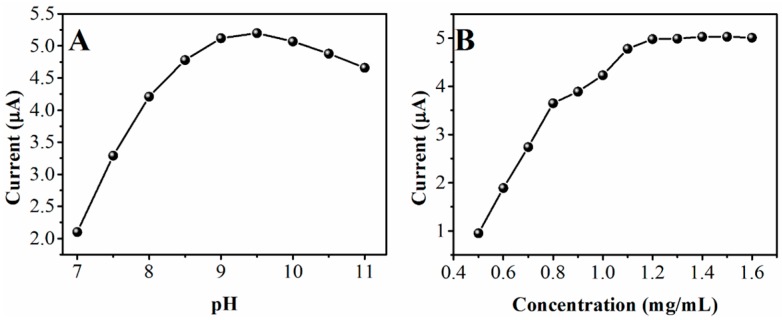
Effect of peak current on (**A**) the pH of the detection solution and (**B**) the concentration of α-naphthyl phosphate (α-NP) at 100 ng/mL NSE.

Moreover, the pH of the substrate solution was an essential factor in the enzyme-catalyzed reaction. As shown in Figure 4A, a maximum response was observed at pH 9.5, where the investigation was performed in the pH range of 7.0 to 11. Hence, a pH of 9.5 was considered to be the optimum value of pH due to the highest activity of AP. Here, the concentration of α-NP in the measurement influenced the behavior of the electrochemical analysis. In Figure 4B, the DPV peak current of the immunosensor in diethanolamine (DEA) increased when the concentration of α-NP increased to 1.6 from 0.5 mg/mL and then reached a plateau when the concentrations were higher. Moreover, the content of the AP-anti-IgG/AuNP-RGO bound on the immunosensor determined the enzymatic reaction rate. Hence, 1.2 mg/mL was fixed as the optimal α-NP concentration to detect AuNP-RGO-amplified DPV.

**Figure 5:**
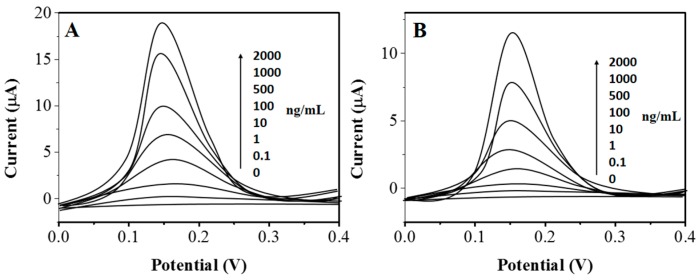
Representative differential pulse voltammetric (DPV) curves of the AP-anti-IgG/AuNP-RGO/anti-NSE/NSE/GCE (**A**) and the alkaline phosphatase (AP)-anti-IgG/anti-NSE/NSE /GCE (**B**) obtained with NSE in a concentration of 0, 0.1, 1, 10, 100, 500, 1000, and 2000 ng/mL.

To evaluate the analytical capacity of the electrochemical immunosensor when the AP-anti-IgG/AuNP-RGO was used as a probe for detection, a control approach was created by employing AP-anti-IgG to bind the anti-NSE/NSE/GCE as a probe in the detection. Figure 5 illustrated the assay of NSE with various level though utilizing diverse probes for detection with the same batch of immunosensors. A control experiment of immunosensor was carried out, where the whole procedure was in the absence of anti-NSE in the first incubation step. No obvious peak was observed in the detection solution. Nevertheless, a significant increase in the DPV signal of blank sample to 15.41 μA was observed when AP-anti-IgG/AuNP-RGO was used as detection probe, where the DPV signal was 6.77 μA by using AP-anti-IgG as detection probe. The obtained results demonstrated that the intensity and the sensitivity of the proposed immunosensor to detect NSE was significantly enhanced by the AP-anti-IgG/AuNP-RGO. A proportional decrease in the DPV peak current was observed under the optimal conditions when the logarithm value of NSE concentration increased. A linear response in the range of 0.1 ng/mL to 0.2 μg/mL was obtained, where the correlation coefficient was 0.989. Moreover, the limit of detection was estimated to be 0.05 ng/mL. The analytical performance of other sensors was utilized as reference to further emphasize the advantages of the proposed electrochemical immunosensor. Compared with Ru(bpy)_3_^2+^-encapsulated silica nanosphere labels [43] and microfluidic immunosensor systems [44], a wider range of detection was exhibited in the designed immunosensor. However, the limit of detection was lower than those of immunosensors modified with the prussian blue-SiO_2_ nanocomposite [45] and the electrochemical catalysis of nickel hexacyanoferrate nanoparticles [46]. By comparison, it demonstrated that the immunosensor exhibited high sensitivity and superiority in the detection of biomarkers with a high concentration. Consequently, the designed approach was effective in directly quantifying the target protein with a wide range of concentration in complicated clinical serum samples.

## 3. Materials and Methods

Neuron specific enolase, AP-labeled goat anti-rabbit antibody (AP-anti-IgG, 1 mg/mL), and rabbit anti-NSE polyclonal antibody (1 mg/mL) were commercially available (Beijing Biosynthesis Biotechnology Ltd. Co., Beijing, China). Graphene oxide was bought from XianfengNano Ltd. Co. (Nanjing, China). HAuCl_4_·4H_2_O was commercially obtained from Sinopharm Chem Ltd. Co. (Shanghai, China). Bovine serum albumin (BSA), chitosan (CS, deacetylation degree was about 90%), *N*-hydroxysuccinimide (NHS), α-naphthyl phosphate (α-NP), and 1-ethyl-3-(3-dimethyllaminopropyl) carbodiimide hydrochloride (EDC) were purchased from Sigma Aldrich (St. Louis, MO, USA). All other reagents were analytically pure unless stated otherwise. Note that all the aqueous solutions were prepared with 18 M ultrapure water.

In a typical procedure of the preparation of the AuNP-RGO, two drops of GO suspension with a concentration of 6 mg/mL was added into 1.4 mL HAuCl_4_ aqueous solution with a concentration of 0.4 mg/mL. Subsequently, 0.4 mL NaOH solution in the concentration of 0.025 mol/L was added dropwise into the mixture. After that, the mixture was stirred with a mechanical stirrer and kept at 82 °C for 15 min. Meanwhile, Au NPs were immobilized onto the surface of the GO nanoplates. Then, 0.02 g CS was added into the stirring mixture, which was subsequently kept stirring at 90 °C for 9 h. At last, the GO nanoplates was reduced to generate the AuNP-RGO.

The GCE was polished to a mirror sheen first with 0.3 and 0.05 μm alumina slurry, respectively, and then rinsed with water. Subsequently, the GCE was sonicated in 1:1 nitric acid and acetone, and deionized water, respectively. After that, the electrode was rinsed again with deionized water and dried under room temperature. Then, the GCE was dipped into the BSA solution (1%) for 30 min to block the nonspecific binding sites on the surface, where the immunosensor was immersed into 50 μL incubation solution. The incubation solution was prepared by mixing 40 μL NSE standard solution or the serum sample with 10 μL anti-NSE primary antibody with a concentration of 5 μg/mL at 37 °C for 40 min for competitive immunoreaction. After that, the immunosensor was rinsed thoroughly with 0.01 M PBS with a pH of 7.4. Then, 5 μL of AP-anti-IgG/AuNP-RGO was deposited on its surface and incubated for 40 min at 37 °C. Followed by rinsing with 0.01 M PBS with a pH of 7.4, the electrochemical test was carried out in the DEA solution with a pH of 9.5 (0.1 mM diethanolamine, 1 mM MgCl_2_, 100 mM KCl) containing 1.2 mg/mL α-NP. The differential pulse voltammetric (DPV) test was carried out with a voltage in the range of 0 to 0.5 V, where the pulse amplitude was 50 mV.

## 4. Conclusions

In conclusion, we improved an electrochemical immunosensor for the determination of NSE at trace levels. Note that all these determinations were carried out on samples which were without any process. This might indicate that the employed antibody was significantly selective. To improve the capacity of the designed immunosensor, an AuNP-RGO composite was prepared. The enhanced immunoreactor could serve as a fast detector with selectivity and sensitivity. Due to the particular reactions between antigens and antibodies, immunosensors was considered as a remarkable alternative for clinical, physiological, and biological tests, and routine veterinary and analytical analyses.
